# Performance of Antibody-Detection Tests for Human Melioidosis: A Systematic Review and Meta-analysis

**DOI:** 10.21315/mjms2024.31.6.4

**Published:** 2024-12-31

**Authors:** Kasturi Selvam, Mohamad Ahmad Najib, Muhammad Fazli Khalid, Azian Harun, Ismail Aziah

**Affiliations:** 1 Institute for Research in Molecular Medicine (INFORMM), Universiti Sains Malaysia, Kelantan, Malaysia; 2 Department of Medical Microbiology and Parasitology, School of Medical Sciences, Universiti Sains Malaysia, Kelantan, Malaysia; 3 Hospital Universiti Sains Malaysia, Kelantan, Malaysia

**Keywords:** melioidosis, Burkholderia pseudomallei, antibody, detection, sensitivity, specificity

## Abstract

Melioidosis is a life-threatening infectious disease caused by the bacterium *Burkholderia pseudomallei*. Although culture is the gold standard for diagnosing melioidosis, it is time-consuming and delays timely treatment. Non-culture-based diagnostic techniques are interesting alternatives for the rapid detection of melioidosis. This systematic review provides an overview of the performance of antibody-detection tests for melioidosis. A thorough literature search was conducted in two databases to identify relevant studies published until 31 December 2023. Among the 453 studies identified, 29 were included for further analysis. Various antibody-detection methods have been developed, primarily enzyme-linked immunosorbent assays (ELISAs). Recombinant outer membrane protein A–(OmpA)-specific immunoglobulin G (IgG), immunoglobulin A (IgA), immunoglobulin M (IgM), and immunoglobulin D (IgD) exhibited the highest accuracy, with a sensitivity of 95.0% and a specificity of 98.0% in ELISA. Furthermore, immunochromatographic testing has emerged as a promising rapid diagnostic test (RDT), with haemolysin co-regulated protein 1 (Hcp1) demonstrating significant accuracy, a sensitivity of 88.3%, and a specificity of 91.6%. Additionally, IgG against *Burkholderia* invasion protein D (BipD) showed excellent accuracy, with a sensitivity of 100.0% and a specificity of 100.0% in surface plasmon resonance assay. Combining multiple antigens or employing different detection techniques can enhance the accuracy of melioidosis diagnosis.

## Introduction

Melioidosis is an infectious disease caused by *Burkholderia pseudomallei*, a gram-negative bacterium found in the soil and groundwater of endemic areas (1, 2). Globally, the burden of human melioidosis is estimated to be 165,000 cases and 89,000 deaths annually. *Burkholderia pseudomallei* is found across the tropics, with Southeast and South Asia, tropical Australia, Western Sub-Saharan Africa, and South America posing the highest risk of infection (3). It is transmitted to humans through the skin (open wounds) via inoculation with contaminated soil or muddy water, the inhalation of contaminated dust or water droplets, or the ingestion of contaminated water or food (4). It is recognised as a great imitator because of its ability to cause a diverse array of clinical symptoms, including pneumonia, skin and soft tissue infections, and internal organ abscess formation (5, 6). Individuals with underlying predisposing medical conditions, particularly diabetes, are considered to be at a high risk for melioidosis (7).

*Burkholderia pseudomallei* is naturally resistant to numerous antibiotics, necessitating a treatment plan of intravenous antibiotic therapy for a minimum of 2 weeks, followed by long-term oral antibiotic therapy (8, 9). Various techniques are used to diagnose melioidosis. Culturing *B. pseudomallei* from clinical specimens, such as blood, pus, and respiratory secretions, is the gold standard. However, this method is time-consuming, has poor sensitivity, and has limited application in endemic regions (10, 11). Since culture techniques have limitations, several researchers have studied alternative diagnostic techniques, including antibody detection. The indirect haemagglutination assay (IHA) is a commonly used method for detecting antibody levels and assessing exposure to *B. pseudomallei*. Nevertheless, it has low sensitivity and specificity, and an inability to track the effectiveness of treatment due to a strong background antibody signal from prior exposure to *B. pseudomallei* and closely related environmental species, such as *Burkholderia thailandensis* (12). Furthermore, the exact antigen used in IHAs is unknown and may differ greatly across laboratories (13).

Despite the unsatisfactory results of IHAs in endemic areas, numerous studies have used various methods and recombinant antigens to assess the accuracy of antibody detection in clinical samples. However, there is a lack of extensive comparisons between these assays, making it difficult to identify appropriate serodiagnostic antigens for *B. pseudomallei*. Therefore, the current review focuses on assessing the performance of developed antibody-detection tests for melioidosis.

## Methods

This review utilised the Preferred Reporting Items for Systematic Reviews and Meta-Analyses (PRISMA) guidelines. The protocol for this review is registered in the PROSPERO database (CRD42023388505).

### Search Strategy

Studies were searched on 31 December 2023 according to the PRISMA guidelines (14). The search was conducted in two databases (PubMed and Scopus) using a list of keywords based on the expanded Medical Subject Headings thesaurus. The keywords were combined using the Boolean operators ‘OR’ and ‘AND’ to generate relevant search results. The search string used was: [“*Burkholderia pseudomallei*” OR “melioidosis”] AND [“antibody”] AND [“diagnosis*” OR “detection*”] AND [“specificity”] AND [“sensitivity”]. An additional search was conducted by manually screening references from the retrieved literature to ensure a comprehensive coverage of relevant studies.

### Selection of Studies

Studies were included in this review if: i) they were cross-sectional, cohort, or case-control studies; ii) antibody-detection tests were conducted on human specimens; iii) they reported diagnostic accuracy metrics, such as sensitivity, specificity, negative predictive value, or positive predictive value, or the data allowed for the calculation of accuracy measures; iv) they used culture as a reference test for melioidosis; v) they were published in English; and vi) they comprised a study population greater than or equal to 10 patients.

Studies were excluded from this review if: i) they were published before 1 January 2000 or after 31 December 2023; ii) they did not report antibody-detection tests for melioidosis; or iii) they were case series or reports, qualitative studies, conference papers, proceedings, abstract-only articles, editorial reviews, letters of communications, commentaries, systematic reviews, or studies of non-living subjects such as soil and water properties.

### Data Extraction and Analysis

The studies were imported into Endnote reference manager (Clarivate, London, UK) and duplicate entries were identified and removed. Two authors (KS and MAN) independently reviewed the titles and abstracts. Satisfactory agreement with the screening process was assessed by the reviewers. Two authors (KS and MFK) performed full-text screening and summarised the findings. The following data were extracted: i) antibody type; ii) detection method; iii) biomarker; iv) human specimen type; v) sample size; vi) specificity; and vii) sensitivity. Two other authors (AH and IA) verified and reviewed the results.

Two authors (KS and MFK) independently collected the number of true positives, true negatives, false positives, and false negatives from each study. Discrepancies were resolved through discussion with a third author (MAN). The sensitivity and specificity of each antibody-detection test were calculated. Sensitivity was calculated by dividing the number of true-positive (TP) results by the total number of true-positive and false-negative (FN) results (Equation 1). Specificity was calculated by dividing the number of true-negative (TN) results by the total number of true-negative and false-positive (FP) results (Equation 2). Performance comparisons of the antibody-detection tests were performed using a forest plot and receiver operating characteristic (ROC) curve with the restricted maximum likelihood method for the random effects model. Heterogeneity between studies was evaluated using Cochran’s Q test with inconsistent values (*I^2^*). An *I^2^* value near 0% indicates no heterogeneity, approximately 25% indicates low heterogeneity, approximately 50% indicates moderate heterogeneity, and approximately 75% indicates high heterogeneity (15). Subgroup meta-analysis was conducted for cases in which high heterogeneity was observed. All statistical analyses were performed using Review Manager (version 5.4.1; Cochrane Collaboration, Copenhagen, Denmark) and the meta (version 7.0.0) package in R (version 4.4.1), as implemented in RStudio (version 2024.04.2-764).


(1) $$sensitivity=\frac{TP}{TP+FN}$$

(2) $$sensitivity=\frac{TP}{TP+FP}$$

### Quality Assessment

The Quality Assessment of Diagnostic Accuracy Studies 2 (QUADAS-2) tool was used to evaluate the quality of each study based on four main domains: patient selection; index test; reference standard; and flow and timing (16). The potential risk of bias (ROB) was assessed for each domain. The authors independently evaluated the quality of each study for all domains, and the ROB was categorised as “low”, “high”, or “unclear”. Three authors (KS, MAN, and MFK) independently assessed the quality of each study. Disagreements between authors were resolved through discussion.

## Results

### Search Results

**Figure 1:**
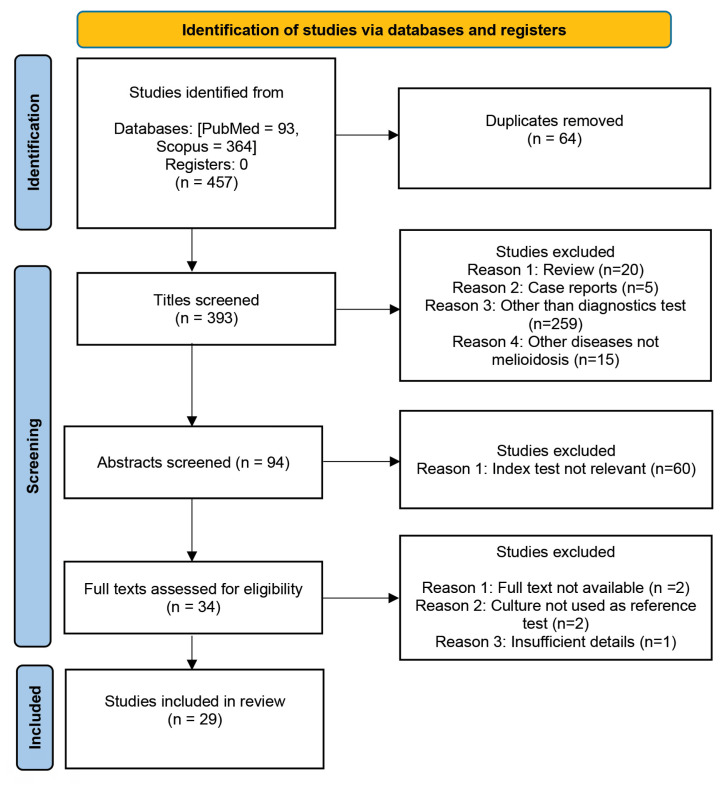
PRISMA flow diagram

A total of 457 studies were identified from the two databases and 64 duplicates were excluded. After screening the titles and abstracts, 359 irrelevant studies were excluded. Five studies were excluded during the full-text screening. The remaining 29 studies were included in the final review (Figure 1). Table 1 summarises the studies that used antibody-detection methods. The total number of studies exceeded 29 because some studies evaluated multiple methods.

### Study Quality

**Figure 2:**
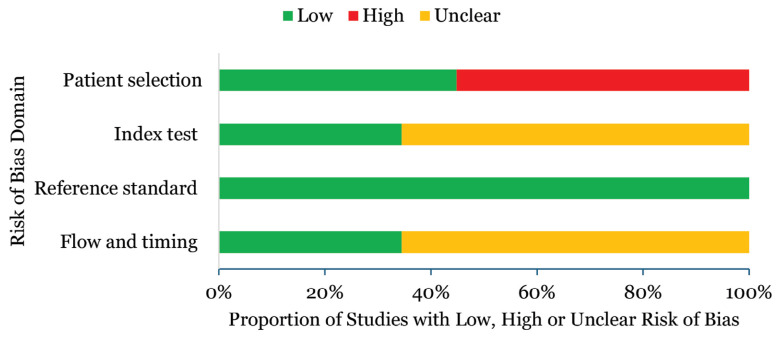
ROB of the included studies Note: Green, yellow, and red colours signify low, unclear, and high risks of bias, respectively

A summary of the QUADAS-2 ROB assessment is shown in Figure 2. Overall, the quality assessment results indicated a high ROB. Regarding patient selection, 16 studies (57.0%) demonstrated a high ROB due to the use of a case-control study design that lacked random participant recruitment and imposed specific criteria for patient selection. For the index test, 19 studies (68.0%) exhibited an unclear ROB, as it remained uncertain whether the index tests were interpreted independently of the reference standard results. All studies demonstrated a low ROB for the reference standard, as they accurately classified patients with culture confirmed melioidosis, without access to the index test results. Regarding flow and timing, 19 studies (68.0%) raised concerns about unclear bias regarding whether all participants, including both the disease and healthy control groups, received the same reference standard.

### Performance of the Antibody-detection Tests

Several antibody-detection methods have been employed to diagnose melioidosis by detecting various classes of immunoglobulins specific to the target antigens, mainly immunoglobulin G (IgG).

### Detection Methods

Various antibody-detection methods have been used to diagnose melioidosis, including the enzyme-linked immunosorbent assay (ELISA), IHA, immunofluorescence antibody test (IFAT), immunochromatographic test (ICT), dot immunoassay (DOT), western blotting, surface plasmon resonance (SPR), latex agglutination (LA), protein microarray, and dipsticks, as shown in Table 1. Multiple studies were conducted using ELISA, IHA, IFAT, ICT, DOT, and western blotting, whereas only a single study was carried out using SPR, LA, protein microarray, and dipstick assays. The overall accuracy of each antibody-detection method varied, with sensitivity ranging from 64.1% to 100.0% and specificity from 70.7% to 100.0%, as detailed in Table 2. Serum samples were predominantly used, except for two studies that used plasma and whole blood samples.

## ELISA

**Figure 3:**
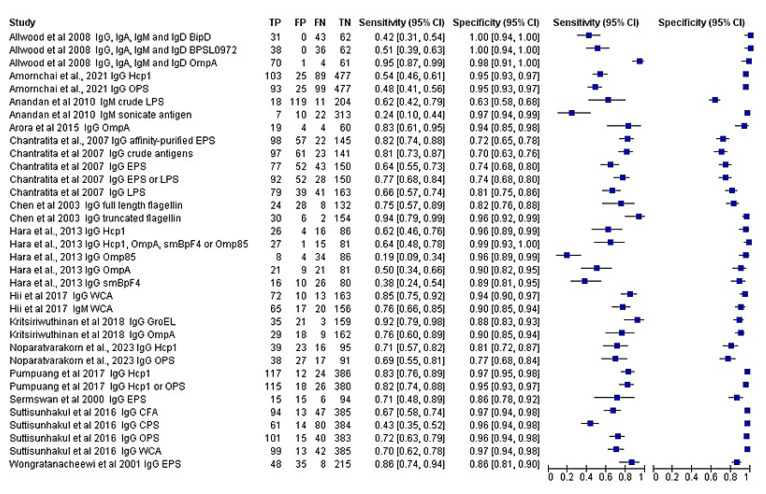
Forest plot analysis of the diagnostic sensitivity and specificity of ELISA-based antibody-detection tests Note: The forest plot represents the estimated sensitivity and specificity (blue squares) and their 95% CIs

In ELISAs, the most commonly used antigen was haemolysin-co-regulated protein (Hcp1), followed by outer membrane protein (OmpA), exopolysaccharide (EPS), and O-polysaccharide (OPS). A meta-analysis of ELISA-based antibody-detection tests revealed varying sensitivities (19.0%–95.0%) and specificities (63.0%–100.0%), as presented in the forest plot (Figure 3). The overall sensitivity and specificity were 67.0% [95% confidence interval (CI): 65.2–68.7] and 89.7% (95% CI: 89.0–90.4), respectively. Heterogeneity of the sensitivity was significant, with a chi-squared (χ^2^) value of 287.2462 (df = 33, *p* < 0.0001) and an *I^2^* value of 91.67%. Similarly, heterogeneity of specificity was significant, with a χ^2^ value of 576.4747 (df = 33, *p* < 0.0001) and an *I^2^* value of 93.72%. The significant heterogeneity observed in both sensitivity and specificity suggested substantial variability across the studies included in the meta-analysis, which may be due to variations in the type of antibody detected and the diversity of antigens utilised.

**Figure 4:**
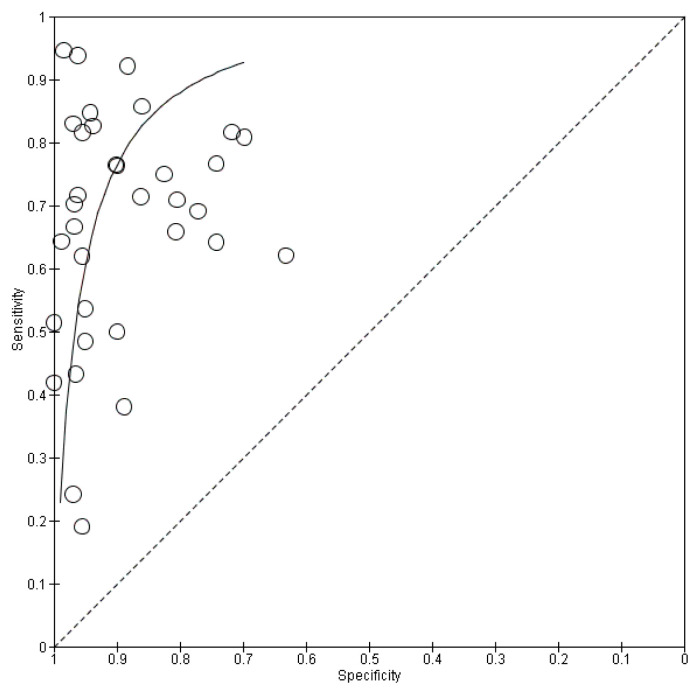
ROC curve indicating the overall performance of ELISA-based antibody-detection tests

Based on the ROC curve (Figure 4), recombinant OmpA-specific IgG, IgA, IgM, and IgD yielded the highest accuracy, with a sensitivity of 95.0% and a specificity of 98.0%. IgG against recombinant truncated flagellin displayed promising accuracy, with a sensitivity of 94.0% and a specificity of 96.0%. Three other studies that detected IgG against recombinant OmpA reported similar specificities (90.0%–94.0%), but varying sensitivities (50.0%–83.0%). Using a multiplex ELISA to detect IgG against recombinant OmpA, along with IgG against three additional antigens (Hcp1, Omp85, and smBpF4), slightly enhanced the sensitivity (64.0%) compared to solely detecting IgG against OmpA (50.0%), while maintaining specificity.

In addition, IgG against Hcp1 demonstrated variable sensitivity between 54.0% and 83.0%, with a high specificity ranging from 81.0% to 97.0%. Its utility extended to other methods, such as IFAT and ICT, where IgG targeting Hcp1 showed sensitivity levels either within or slightly surpassing the range observed in the ELISAs. OPS-specific IgG revealed lower sensitivity (ranging from 48.0% to 72.0%), but higher specificity (ranging from 77.0% to 96.0%). Combining IgG specific for OPS and Hcp1 resulted in improved sensitivity (82.0%), and maintained good specificity (96.0%) compared to IgG specific OPS alone.

Detection of IgG, IgA, IgM, and IgD antibodies against recombinant *Burkholderia* invasion protein D (BipD) revealed a sensitivity of 42.0%, but achieved the highest specificity of 100.0%. In contrast, western blotting analysis using IgG antibodies targeting recombinant BipD (with the GST tag removed) exhibited a robust sensitivity of 100.0%, albeit with a slightly lower specificity of 91.0%. Furthermore, SPR demonstrated unparalleled sensitivity and specificity, both at a perfect 100.0% for BipD-specific IgG. Additionally, IgG antibodies against culture filtrate antigens (CFAs) yielded a comparably low sensitivity of 67.0%, which was similar to the overall sensitivity observed for IHA using CFAs (64.0%; Table 2).

## Other Methods

In IHA studies, CFAs were exclusively utilised, yielding an overall sensitivity of 64.1% and a specificity of 82.5%. IFAT-based studies used whole-cell antigens from *B. pseudomallei* and *B. thailandensis* or recombinant proteins expressed in *E. coli*. In the majority of the ICT-based studies, the Melioidosis Rapid Cassette Test kit produced by Pan-Bio (Windsor, Queensland, Australia) was used, accounting for 75% of the studies in which the specific antigen used was not disclosed.

In the DOT-based studies, two antigens (CFA and GroEL) were used. Western blotting studies employed two antigens, Bps-1 and BipD. The BipD was also used for SPR and showed excellent accuracy (sensitivity: 100.0% and specificity: 100.0%). Next, LA employs two antigens, OPS and capsular polysaccharide (CPS), both of which have poor specificity.

Thirteen serodiagnostic protein markers were identified using a protein microarray, with three of them, namely OmpA (BPSL2522), GroEL, and hydroperoxide reductase (AhpC, BPSL2096), being employed in other detection methods. Hcp1, GroEL1, GroEL2, and AhpC were utilised in four-plex dipstick assays, yielding promising accuracy with a sensitivity of 92.0% and a specificity of 97.0%.

### Subgroup Meta-analysis

**Figure 5:**
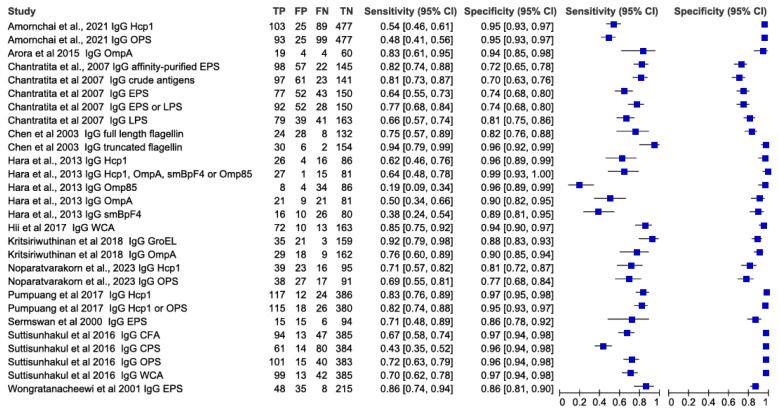
Forest plot analysis of the diagnostic sensitivity and specificity of IgG ELISA-based antibody-detection tests Note: The forest plot represents the estimated sensitivity and specificity (blue squares) and their 95% CIs

**Figure 6:**
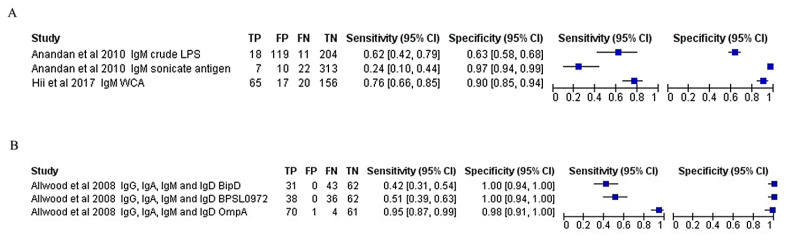
Forest plot analysis of the diagnostic sensitivity and specificity of (A) IgM and (B) total antibody ELISA-based antibody-detection tests Note: The forest plot represents the estimated sensitivity and specificity (blue squares) and their 95% CIs

**Figure 7:**
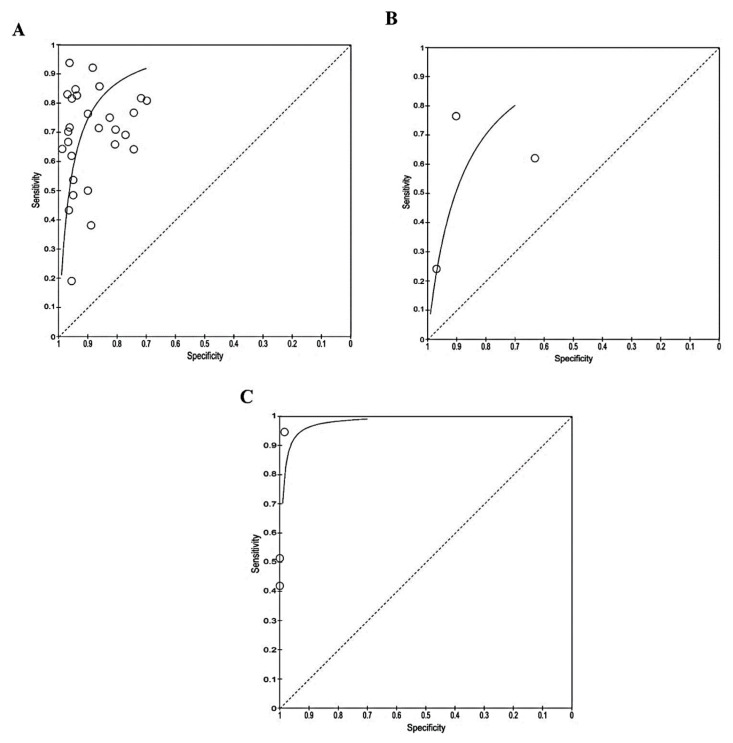
ROC curve indicating the overall performance of (A) IgG, (B) IgM, and (C) total antibody ELISA-based antibody-detection tests

**Figure 8:**
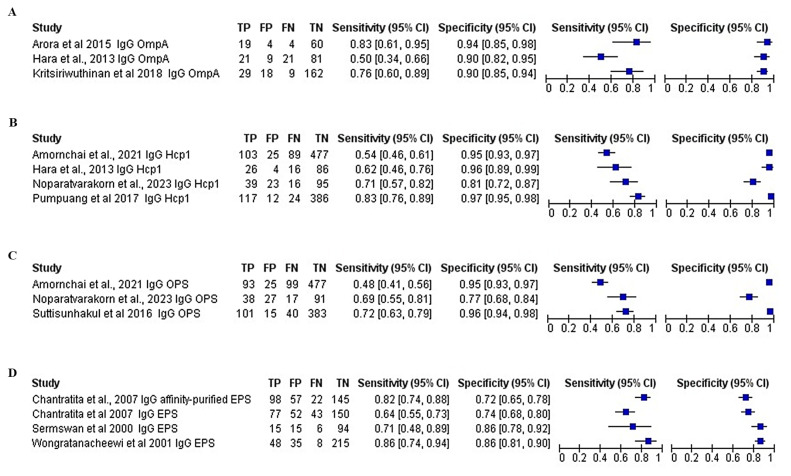
Forest plot analysis of the diagnostic sensitivity and specificity of (A) OmpA, (B) Hcp1, (C) OPS, and (D) EPS ELISA-based antibody-detection tests Note: The forest plot represents the estimated sensitivity and specificity (blue squares) and their 95% CIs

**Figure 9:**
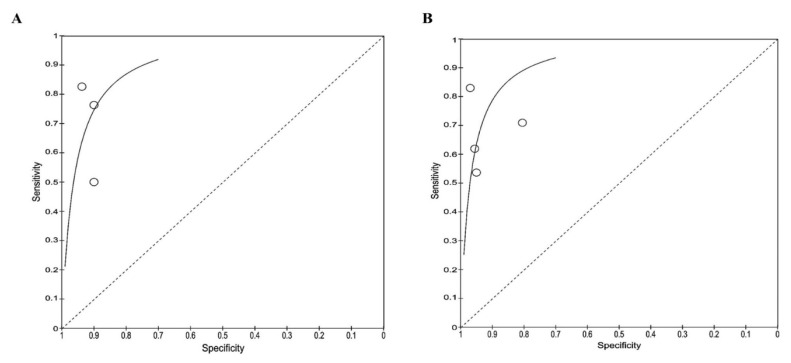
The ROC curve that indicates the overall performance of (A) OmpA and (B) Hcp1 based antibody-detection tests

**Figure 10:**
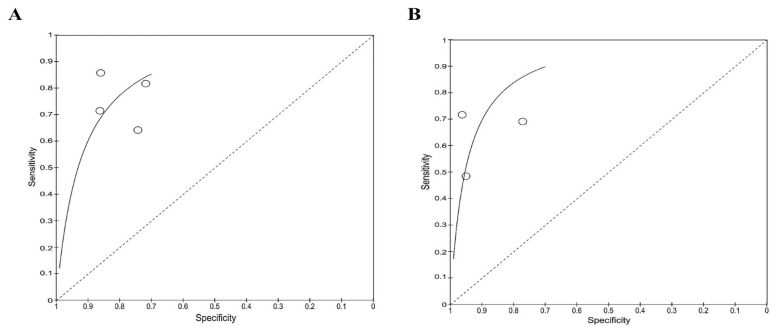
ROC curve indicating the overall performance of (A) EPS- and (B) OPS-based antibody-detection tests

High heterogeneity was observed for both sensitivity and specificity for the ELISA-based antibody-detection tests. Therefore, subgroup analysis was performed based on the type of antibody detected, such as IgG ELISA, IgM ELISA, and total antibody (IgG ELISA, IgM ELISA, IgA, and IgD), as presented in the forest plots (Figure 5–6) and ROC curve (Figure 7). Another subgroup analysis was performed based on the use of antigens, such as OmpA, Hcp1, OPS, and EPS, as presented in the forest plot (Figure 8) and ROC curve (Figure 9–10). A summary of the subgroup analyses is presented in Table 3.

The overall sensitivity of the total antibody ELISA demonstrated an *I^2^* value of 97.01%, indicating high heterogeneity, and the overall specificity showed an *I^2^* value of 0%, suggesting no heterogeneity. A similar pattern was observed for the OmpA ELISA, for which the overall sensitivity had an *I^2^* value of 76.49%, reflecting high heterogeneity, and the overall specificity had an *I^2^* value of 0%, indicating no heterogeneity. However, it is important to note that these subgroup meta-analyses included only three studies.

## Discussion

Melioidosis has a higher burden worldwide than other widely recognised diseases, such as leptospirosis, dengue schistosomiasis, lymphatic filariasis, and leishmaniasis (46). The gold standard for diagnosing melioidosis is the culture of *B. pseudomallei* from clinical samples (47). The performance and turnaround time of diagnostic tests are crucial for effective management of melioidosis. Non-culture-based diagnostic tests are needed to achieve the timely initiation of antibiotic therapy. Currently, no commercial rapid diagnostic tests (RDTs) are available as alternatives to the current reference standard tests for melioidosis.

The InBiOS Active Melioidosis Detect (AMD) Rapid Test Kit employs a lateral flow immunoassay to detect CPS of *B. pseudomallei* using a CPS-specific monoclonal antibody. This test strip is not commercially available yet and is only used for research purposes. The sensitivity of this assay was found to be lower in whole blood, serum, and plasma (17.0%–25.0%) than in other sample types, such as urine, pus, and sputum (48–50). In contrast, molecular methods such as polymerase chain reaction (PCR) and quantitative PCR (qPCR), have been conducted using various genes, particularly the type III secretion system gene cluster (TTS1). These methods offer enhanced sensitivity, but their application requires the isolation of bacterial DNA, specialised equipment, stringent handling procedures, and expert operators (30).

In addition, antibody-detection methods have been widely studied for the diagnosis of melioidosis, but their utility is limited by high rates of background seropositivity in endemic areas, making it challenging to distinguish between acute and convalescent cases (12). Despite these challenges, serological ELISA, ICT, IFA, and other methods using various antigens have been conducted with a broad range of reported sensitivities and specificities in addition to IHA. Therefore, the present systematic review sought to evaluate the performance of antibody-detection tests for melioidosis reported in the past 23 years.

This review revealed that ELISA is the primary method used to detect antibodies for the diagnosis of melioidosis. ELISA-based antibody tests have been standardised using a microplate reader and recombinant proteins, reducing interlaboratory variation compared to the IHA test which has variations in CFA preparation between laboratories and produces inconsistent results between observers (47, 51). A meta-analysis was conducted to evaluate ELISA-based antibody-detection methods. Recombinant OmpA-specific IgG, IgA, IgM, and IgD exhibited superior diagnostic performance in distinguishing melioidosis cases from cases of other infections and healthy individuals. This effectiveness may greatly facilitate clinical decision-making by minimising the occurrence of false-positive results attributed to other infections (21). Moreover, one study reported that OmpA is a useful marker for detecting previous, but not recent, infections because it can recognise sera from weeks 0 to 52 post-admission (44). Three additional studies assessing OmpA-specific IgG reported lower sensitivity. This discrepancy may be attributed to the fact that one study detected total antibodies, while another study focused solely on one antibody type, as well as variations in the employed cut-off values (23, 24, 28).

Furthermore, IgG against recombinant truncated flagellin resulted in higher sensitivity and specificity than IgG against recombinant full-length flagellin. The amino acid sequence of flagellin was similar to those of *Pseudomonas aeruginosa*, *Salmonella enterica serovar* Typhimurium, *Proteus mirabilis*, and *Escherichia coli* at the N- and C-termini (1–40 and 300–387, respectively). Consequently, truncated flagellin, synthesised from amino acids 41–299, has been employed to minimise cross-reactivity with other bacteria, leading to an increase in test sensitivity (19). In addition, truncated flagellin from *B. thailandensis*, known as FLAG300 was employed in ELISAs for melioidosis antibody detection and resulted in a slight decrease in accuracy compared to that of truncated flagellin from *B. pseudomallei*, with a sensitivity of 82.7%–90.48% and a specificity of 87.14%–94.6% (52, 53).

Hcp1 is the most commonly utilised antigen in ELISAs, possibly because of its structural dissimilarity to Hcp1 of *B. thailandensis*, which may aid in reducing background antibody levels among healthy donors in endemic regions (27). Additionally, Hcp1 can bind to the surface of host antigen-presenting cells, potentially enhancing their immunogenicity and prompting stronger antibody responses in individuals with melioidosis (54). Discrepancies in the timing of sample collection may account for the variability in sensitivity observed across different studies. Furthermore, *B. pseudomallei* OPS can be classified as typical type A, atypical types B1 and B2, or rough variants (55). The OPS used in the included studies was derived from *B. pseudomallei* LPS type A, potentially limiting sensitivity in cases where patients are infected with *B. pseudomallei* featuring atypical or rough LPS types.

The IHA remains a widely used serological test for clinical epidemiology and case detection because of its low cost and ease of application (13). In this review, all IHA studies used CFA to detect total antibodies against *B. pseudomallei* and showed a low overall sensitivity of 64.1%. Based on the included studies, different cut-off values have been used in IHAs, such as ≥ 1:20, ≥ 1:40, and ≥ 1:160, depending on the country and its endemicity level. In IFAT, IgG and IgM of *B. thailandensis* showed comparable sensitivity and specificity compared to *B. pseudomallei* (38). *Burkholderia thailandensis* is closely related to *B. pseudomallei* and is generally considered nonpathogenic to humans. Due to the many genetic and phenotypic characteristics and similarities between the two species, *B. thailandensis* is often used as a model organism to study *B. pseudomallei* (56, 57).

Most ICT-based studies have used the Melioidosis Rapid Cassette Test kit (Pan-Bio), which is not commercially available. Despite its advantages, such as providing rapid results and ease of use, it demonstrated unsatisfactory accuracy in diagnosing melioidosis, whether detecting IgM, IgG, or both (39). Conversely, the use of Hcp1 in ICT demonstrated superior accuracy compared to the Melioidosis Rapid Cassette Test kit, with a sensitivity of 88.3% and a specificity of 91.6%. Of the patients initially showing negative culture results, 31% who subsequently tested positive for the Hcp1-ICT were later confirmed to have *B. pseudomallei* infection through culture. Given its rapid 15-minute turnaround time, the Hcp1-ICT may prompt clinicians to consider testing for melioidosis in patients with unknown infections (30, 40).

BipD is a needle-tip protein of the type III secretion system. It assists *B. pseudomallei* in invading nonphagocytic cells, escaping from the phagosome, and promoting intracellular replication (58–60). Interestingly, a western-blotting-based study showed that IgG against recombinant BipD had higher sensitivity and specificity than IgG against recombinant GST-BipD. This result revealed that the sensitivity of the test increased after GST was removed, possibly because the presence of GST hindered the binding of BipD to the antibodies (42). BipD has also been utilised in other antibody-detection methods exhibiting high specificity and sensitivity, with the exception of ELISAs. This finding suggests that BipD is specific to *B. pseudomallei* and is less similar to homologous proteins found in different bacteria (e.g., *Salmonella* invasion protein D (SipD) from *Salmonella*: 26.0% identity, 36.0% similarity and Invasion plasmid antigen D (IpaD) from *Shigella*: 27.0% identity, 39.0% similarity) (61). The sensitivity of BipD-specific IgG can be enhanced by employing highly sensitive detection methods, such as SPR (43). BipD is a potential biomarker for identifying sera from healthy individuals with melioidosis (62).

This review also highlights a four-plex dipstick with promising accuracy for diagnosing melioidosis (sensitivity, 92.0%; specificity, 97.0%). The dipstick assay accelerates the diagnostic process to just 15 minutes, reduces expenses associated with more intricate laboratory methods, and is beneficial in resource-limited settings (45). Small proteins are recommended for multiplex detection, because larger proteins may overshadow smaller ones (23). Moreover, combining these two techniques may enhance the detection of melioidosis. The combination of Hcp1-ICT (antibody detection) with TTS1-PCR (real-time PCR test based on type 3 secretion system 1 genes) significantly improved sensitivity from 74.5% (Hcp1-ICT) to 98.2% (combination), without compromising assay specificity (30). Additionally, combining antigen detection (CPS-Lateral flow immunoassay) with antibody detection (Hcp1-ELISA or OPS-ELISA) increased the sensitivity compared to any single test, while maintaining high specificity (95.0%) (29). Therefore, future studies should prioritise the development of multiplex tests (using multiple antigens or combining different techniques) to more effectively identify patients with melioidosis.

This study has several limitations. First, most studies included in the meta-analysis were case-control studies, which may be less representative of clinical practice, although they may be easier to conduct in laboratory settings than cross-sectional designs. It is important to note that the performance of diagnostic tests may vary depending on the population in which they are used (63). Second, this study only included articles written in English, which may have introduced selection bias into the results (64). Third, publication bias may have resulted in an overestimation of diagnostic performance. For example, studies with poor diagnostic performance are unlikely to be published (65). Publication bias, also known as reporting bias, is widely recognised, wherein the nature and direction of results influence the decision to publish relevant trials. Studies with significant results were more likely to be published (66). Fourth, high heterogeneity was observed in the ELISA-based antibody-detection tests. Therefore, this study conducted a subgroup meta-analysis. These findings indicate that the reliability of both total antibody and OmpA ELISAs in identifying true negatives is consistent across different settings or populations. However, the ability of these tests to accurately identify individuals with melioidosis varies significantly across studies, which may be attributed to factors such as the immunogenicity of the antigens, the severity of the infection, the endemic nature of the regions, and the sample size. Finally, the analysis of antibody-detection performance may have been affected by the suboptimal sensitivity of the culture method. Culture is acknowledged as an imperfect reference standard that poses the risk of false-negative outcomes (11). Consequently, false-negative culture results can lead to decreased specificity of serological tests (index tests).

## Conclusion

This systematic review offers an overview of antibody-detection tests for melioidosis and highlights key findings. First, ELISA has emerged as the predominant method for melioidosis serodiagnosis because of its standardisation and its ability to reduce interlaboratory variance compared with IHA. Nonetheless, the utility of ELISA in on-site applications is limited compared to that of ICT, which is faster and can be utilised by the general public as end users. Secondly, several antigens, including OmpA, Hcp1, and BipD, have shown promising sensitivity and specificity for detecting *B. pseudomallei*-specific antibodies, although they require further comprehensive evaluation. These three antigens appear to be suitable options for the development of point-of-care RDT for the diagnosis of melioidosis. Third, the accuracy of antibody-detection tests can be enhanced through antigen truncation (e.g., flagellin) or the removal of large tags (e.g., GST). Furthermore, combining multiple antigens in a single technique or employing different detection techniques can enhance the accuracy of melioidosis detection.

## Figures and Tables

**Table 1:** Characteristics of the included studies based on the antibody-detection method

No.	Study	Type of antibody	Biomarker	Type of sample	Number of samples (n)	Sensitivity (%)	Specificity (%)
**ELISA**							

1	Sermswan et al. (17)	IgG	Immunoaffinity-purified EPS	Serum	Culture confirmed *Bp* (n = 21) DC (n = 109)	71.4	86.2

2	Wongratanacheewi et al. (18)	IgG	Immunoaffinity-purified EPS	Serum	Culture confirmed *Bp* (n = 56) DC (n = 250)	85.7	86.0

3	Chen et al. (19)	IgG	Recombinant full-length flagellin	Serum	Culture confirmed *Bp* (n = 32) DC (n = 100) HC [Taiwan] (n = 60)	75.0	82.5
			Recombinant truncated flagellin	Serum		93.8	96.3

4	Chantratita et al. (20)	IgG	Affinity-purified EPS	Serum	Culture confirmed *Bp* (n = 120) DC (n = 202)	82.0	72.0
			Crude antigens			81.0	70.0
			Purified EPS and LPS			77.0	74.0
			Purified LPS			66.0	81.0
			Purified EPS			64.0	74.0

5	Allwood et al. (21)	IgG, IgA, IgM, and IgD	Recombinant BPSL0972	Serum	Culture confirmed *Bp* (n = 74) DC (n = 20) HC (n = 42) -endemic [Thailand] (n = 18) -non-endemic [Queensland, Australia] (n = 24)	51.0	100.0
			Recombinant BipD			42.0	100.0
			Recombinant OmpA			95.0	98.0

6	Anandan et al. (22)	IgM	Sonicate antigen	Serum	Culture confirmed *Bp* (n = 29) DC (n = 214) HC [India] (n = 109)	25.0	96.8
			Crude LPS	Serum		62.0	63.3

7	Hara et al. (23)	IgG	Recombinant TssD-5 (Hcp1)	Serum	Culture confirmed *Bp* (n = 42) DC (n = 29) HC [Malaysia] (n = 61)	62.0	96.0
			Recombinant Omp3 (OmpA)			50.0	90.0
			Recombinant smBpF4			38.0	89.0
			Recombinant Omp85			19.0	96.0
			Recombinant antigens (Hcp1, OmpA, smBpF4, and Omp85)			64.0	99.0

8	Arora et al. (24)	IgG	Recombinant OmpA	Serum	Culture confirmed *Bp* (n = 23) DC (n = 25) HC [India] (n = 39)	82.6	93.8

9	Suttisunhakul et al. (25)	IgG	Purified OPS	Serum	Culture confirmed *Bp* (n = 141) DC (n = 120) HC (n = 278) -endemic [Thailand] (n = 188) -non-endemic [U.S] (n = 90)	71.6	96.2
			WCA			70.2	96.7
			CFA			66.7	96.7
			CPS Purified	Serum		43.5	96.5

10	Hii et al. (26)	IgG	WCA	Serum	Culture confirmed *Bp* (n = 85) DC (n = 65) HC [Malaysia] (n = 108)	84.7	93.6
		IgM	WCA			76.1	90.2

11	Pumpuang et al. (27)	IgG	Recombinant Hcp1	Serum	Culture confirmed *Bp* (n = 141) DC (n = 120) HC (n = 278) -endemic [Thailand] (n = 188) -non-endemic [US] (n = 90)	83.0	97.0
			Hcp1 or OPS			81.6	95.5

12	Kritsiriwuthinan et al. (28)	IgG	Recombinant OmpA	Serum	Culture confirmed *Bp* (n = 38) DC (n = 55) HC [Thailand] (n = 125) -endemic (n = 77) -non-endemic (n = 48)	76.0	90.0
			Recombinant GroEL			92.0	88.0

13	Amornchai et al. (29)	IgG	Recombinant Hcp1	Serum	Culture confirmed *Bp* (n = 192) DC (n = 502)	53.6	95.0
			Recombinant OPS			48.4	95.0

14	Noparatvarakorn et al. (30)	IgG	Recombinant Hcp1	Plasma	Culture confirmed *Bp* (n = 55) DC (n = 49) HC [Thailand] (n = 69)	70.9	80.5
			Recombinant OPS			69.1	77.1

**IHA**							

1	Sermswan et al. (17)	Total Ab	CFA	Serum	Culture confirmed *Bp* (n = 21) DC (n = 109)	61.9	79.8

2	Wongratanacheewi et al. (18)	Total Ab	CFA	Serum	Culture confirmed *Bp* (n = 56) DC (n = 250)	50.0	72.0

3	O’Brien et al. (31)	Total Ab	CFA	Serum	Culture confirmed *Bp* (n = 10) DC (n = 150)	90.0	91.3

4	Chuah et al. (32)	Total Ab	CFA	Serum	Culture confirmed *Bp* (n = 75) DC (n = 45) HC [North Queensland, Australia] (n = 113)	76.0	99.1

5	Chantratita et al. (20)	Total Ab	CFA	Serum	Culture confirmed *Bp* (n = 120) DC (n = 202)	73.0	64.0

6	Suttisunhakul et al. (33)	Total Ab		Serum	Culture confirmed *Bp* (n = 141) HC (n = 278) [Thailand] (n = 188) [US] (n = 90)	69.5	78.1

7	Kritsiriwuthinan et al. (34)	Total Ab	CFA	Serum	Culture confirmed *Bp* (n = 42) DC (n = 74) HC [Thailand] (n=175)	64.3	85.5

8	Lantong et al. (35)	Total Ab	CFA	Serum	Culture confirmed *Bp* (n = 81) DC (n = 70) HC [Thailand] (n = 120)	37.0	99.5

**IFAT**							

1	Vadivelu and Puthucheary (36)	IgG and IgM	WCA	Serum	Culture confirmed *Bp* (n = 66) DC (n = 523)	100.0	71.5

2	Mathai et al. (37)	IgM	WCA	Serum	Culture confirmed *Bp* (n = 22) DC (n = 208) HC [India] (n = 108)	59.0	94.3
		IgG				45.5	94.3
		IgG and IgM				36.0	99.1

3	Puthucheary et al. (38)	IgG and IgM	WCA from *B. pseudomallei*	Serum	Culture confirmed *Bp* (n = 12) DC (n = 46) HC [Malaysia] (n = 50)	100.0	96.9
			WCA from *B. thailandensis*			100.0	95.8

4	Lantong et al. (35)	IgG	Recombinant protein expressing *E. coli* (WCA) TssM	Serum	Culture confirmed *Bp* (n = 81) DC (n = 70) HC [Thailand] (n = 120)	92.6	100.0
			OmpH			88.9	100.0
			AhpC			85.2	100.0
			BimA			79.0	100.0
			Hcp1			61.7	100.0

**ICT**							

1	O’Brien et al. (31)	IgG	NR	Serum	Culture confirmed *Bp* (n = 10) DC (n = 150)	70.0	90.0
		IgM				100.0	68.7

2	Chuah et al. (32)	IgG	NR	Serum	Culture confirmed *Bp* (n = 75) DC (n = 45) HC [North Queensland, Australia] (n = 113)	50.6	97.4
		IgM				72.0	71.5

3	Cheng et al. (39)	IgG	NR	Serum	Culture confirmed *Bp* (n = 120) DC (n = 202)	88.0	48.0
		IgM				82.0	47.0
		IgG and IgM				78.0	62.0
		IgG or IgM				92.0	32.0

4	Phokrai et al. (40)	IgG	Recombinant Hcp1	Serum	Culture confirmed *Bp* (n = 487) DC (n = 207) HC (n = 292) -endemic [Thailand] (n = 202) -non-endemic [US] (n = 90)	88.3	91.6

5	Noparatvarakorn et al. (30)	IgG	Recombinant Hcp1	Whole blood	Culture confirmed *Bp* (n = 55) DC (n = 49) HC [Thailand] (n = 69)	74.5	83.9

**DOT**							

1	Sermswan et al. (17)	IgG, IgM, and IgA	CFA	Serum	Culture confirmed *Bp* (n = 21) DC (n = 109)	85.7	85.3

2	Wongratanacheewi et al. (18)	IgG, IgM, and IgA	CFA	Serum	Culture confirmed *Bp* (n = 56) DC (n = 250)	96.4	84.0

3	Kritsiriwuthinan et al. (34)	IgG	Recombinant GroEL	Serum	Culture confirmed *Bp* (n = 42) DC (n = 74) HC [Thailand] (n = 175)	85.7	94.4

**Western blot**							

1	Wongprompitak et al. (41)	Total Ab	Recombinant Bps-I	Serum	Culture confirmed *Bp* (n = 76) -septicaemic (n = 46) -localised (n = 30) DC (n = 75) HC (n = 232) -endemic [Thailand] (n = 132) -non-endemic [Bangkok] (n = 100)	69.7	96.4

2	Visutthi et al. (42)	IgG	Recombinant BipD	Serum	Culture confirmed *Bp* (n = 27) DC (n = 65) HC (n = 25)	100.0	91.1
			Recombinant GST- BipD			78.0	90.0

**SPR**							

1	Dawan et al. (43)	Total Ab	Recombinant BipD	Serum	Culture confirmed *Bp* (n = 20) DC (n = 20) HC [Thailand] (n = 20)	100.0	100.0

**LA**							

1	Suttisunhakul et al. (33)	Total Ab	Purified OPS	Serum	Culture confirmed *Bp* (n = 141) HC (n = 278) [Thailand] (n = 188) [US] (n = 90)	84.4	70.1
			CPS Purified	Serum		69.5	74.8

**Protein Microarray**							

1	Kohler et al. (44)	IgG	Recombinant 20 antigens (FlgK, BPSL1445, BPSL1661 (1001), BPSL1661 (1002), BPSL2030, BPSL2096, BPSL2520, BPSL2522, GroEL, GroES, BPSL3319, BPSS0476, BPSS0477, BPSS0530, BPSS1385, BPSS1516, BPSS1525, BPSS1532, BPSS1722, BPSS2141) [NI>0.3]	Serum	Culture confirmed *Bp*(n = 171) -week 0 (n = 75) -week 12 (n = 50) -week 52 (n = 46) DC (n = 60) HC (n = 125) -endemic and non- endemic [Thailand] (n = 100) -non-endemic [Germany] (n = 25)	86.7 (week 0) 82.0 (week 12) 56.5 (week 52)	97.0

**4-plex dipstick**							

1	Wagner et al. (45)	4-plex dipstick [IgG]	Recombinant antigens (AhpC, GroEL1, GroEL2, and Hcp1)	Serum	Culture confirmed *Bp* (n = 75) DC (n = 60) HC [Thailand] (n = 100) -endemic (n = 75) -non-endemic (n = 25)	92.0	97.0

Notes: IHA: indirect haemagglutination assay; Ab: antibody; CFA: culture filtrate antigen; DC: febrile patients with other bacterial, fungal, or viral infections and pyrexia of unknown origin; HC: healthy donors or individuals; IFAT: immunofluorescence antibody test; IgG: immunoglobulin G; IgM: immunoglobulin M; WCA: whole-cell antigens; Hcp1 (TssD-5): haemolysin co-regulated protein 1; AhpC: hydroperoxide reductase; TssM: type VI secretion system protein M; OmpH: outer membrane protein H; BimA: *Burkholderia* intracellular motility factor A; ICT: immunochromatographic test; NR: not reported; DOT: dot immunoassay; IgA: immunoglobulin A; GroEL: molecular chaperone; BipD: *Burkholderia* invasion protein D; ELISA: enzyme-linked immunosorbent assay; EPS: exopolysaccharide; LPS: lipopolysaccharide; OmpA (Omp3): outer membrane protein A; smBpF4: serine protease; Omp85: outer membrane protein 85; OPS: O-polysaccharide; CPS: capsular polysaccharide; BPSL0972: putative exported protein; SPR: surface plasmon resonance; LA: latex agglutination; FlgK: flagellar hook-associated protein; BPSL1445: putative lipoprotein; BPSL1661: putative haemolysin-related protein; BPSL1661: putative haemolysin-related protein; BPSL2030: putative exported protein; BPSL2096: AhpC; BPSL2520: putative exported protein; BPSL2522: OmpA; GroES: molecular chaperone; BPSL3319: flagellin;BPSS0476: molecular chaperone; BPSS0477: molecular chaperone; BPSS0530: conserved hypothetical protein; BPSS1385: ATP/GTP binding protein; BPSS1516: effector protein; BPSS1525: G-nucleotide exchange factor; BPSS1532: putative cell invasion protein; BPSS1722: malate dehydrogenase; BPSS2141: periplasmic oligopeptide-binding protein precursor; NI: normalized intensities; GroEL1: molecular chaperone GroEL1; GroEL2: molecular chaperone GroEL2; GST: glutathione S-transferase; Bps-1: 18.7 kDa protein; Bp: *Burkholderia pseudomallei*

**Table 2:** Summary of the accuracy of antibody-detection methods and immunoglobulin classes

Serological method	Immunoglobulin class	Number of studies*	TP	FP	FN	TN	Sensitivity (%) [95% CI]	Specificity (%) [95% CI]
ELISA	Overall	34	1,902	758	938	6,603	67.0 [65.2–68.7]	89.7 [89.0–90.4]
	IgG	28	1,673	611	802	5,745	67.6 [65.8–69.4]	90.4 [89.7–91.1]
	IgM	3	81	146	62	673	56.6 [48.5–64.8]	82.2 [79.6–84.8]
	IgG, IgA, IgM, and IgD	3	139	1	83	185	62.6 [56.2–69.0]	99.5 [98.4–100.0]

IHA	Overall [total Ab]	8	350	277	196	1,309	64.1 [60.0–68.1]	82.5 [80.7–84.4]

IFAT	Overall	11	451	195	110	2,418	80.4 [77.1–83.7]	92.5 [91.5–93.5]
	IgG	6	340	18	87	1,248	79.6 [75.8–83.4]	98.6 [97.9–99.2]
	IgM	1	13	18	9	298	59.1 [38.5–79.6]	94.3 [91.7–96.9]
	IgG and IgM	4	98	159	14	872	87.5 [81.4–93.6]	84.6 [82.4–86.8]

ICT	Overall	10	988	598	204	1,443	82.9 [80.7–85.0]	70.7 [68.7–72.7]
	IgG	5	622	185	125	942	83.3 [80.6–85.9]	83.6 [81.4–85.7]
	IgM	3	162	199	43	311	79.0 [73.5–84.6]	61.0 [56.7–65.2]
	IgG and IgM	1	94	77	26	125	78.3 [71.0–85.7]	61.8 [55.2–68.6]
	IgG or IgM	1	110	137	10	65	91.7 [86.7–96.6]	32.2 [25.7–38.6]

DOT	Overall	3	108	70	11	538	90.8 [85.6–96.0]	88.5 [86.0–91.0]
	IgG	1	36	13	6	236	85.7 [75.1–96.3]	94.8 [92.0–97.5]
	IgG, IgM, and IgA	2	72	57	5	302	93.5 [88.0–99.0]	84.1 [80.3–87.9]

Western blot	Overall	3	101	28	29	459	77.7 [70.5–84.8]	94.2 [92.1–96.3]
	IgG	2	48	17	6	163	88.9 [80.5–97.3]	90.6 [86.3–94.8]
	Total Ab	1	53	11	23	296	69.7 [59.4–80.1]	96.4 [94.3–98.5]

SPR	Overall [total Ab]	1	20	0	0	40	100.0 [100.0–100.0]	100.0 [100.0–100.0]

LA	Overall [total Ab]	2	217	153	65	403	77.0 [72.0–81.9]	72.5 [68.8–76.2]

Protein microarray	Overall [IgG]	1	65	4	10	121	86.7 [79.0–94.3]	96.8 [93.7–99.9]

4-plex dipstick	Overall [IgG]	1	69	3	6	157	92.0 [85.9–98.1]	98.1 [96.0–100.0]

* The sum of the number of studies exceeded 28 because some studies evaluated more than one antibody-detection method and immunoglobulin class.

TP: true-positive; FP: false-positive; TN: true-negative; FN: false-negative; ELISA: enzyme-linked immunosorbent assay; IgG: immunoglobulin G; IgM: immunoglobulin M; IgA: immunoglobulin A; IgD: immunoglobulin D; Ab: antibody; IHA: indirect haemagglutination assay; IFAT: immunofluorescence antibody test; ICT: immunochromatographic test; DOT: dot immunoassay; SPR: surface plasmon resonance; LA: latex agglutination; Cl: confidence interval

**Table 3:** Summary of subgroup meta-analysis and heterogeneity

Subgroup of ELISA	Number of studies	Overall sensitivity % (95% CI)	Overall specificity % (95% CI)	Heterogeneity of sensitivity % (*p*-value)	Heterogeneity of specificity % (*p*-value)
Type of antibody detected					
IgG	28	67.6 (65.8–69.4)	90.4 (89.7–91.1)	90.19 (<0.0001)	93.17 (<0.0001)
IgM	3	56.6 (48.5–64.8)	82.2 (79.6–84.8)	91.15 (<0.0001)	97.68 (<0.0001)
Total antibody	3	62.6 (56.2–69.0)	99.5 (98.4–100.0)	97.01 (<0.0001)	0.0 (0.9974)

Type of antigen utilised					
OmpA	3	67.0 (57.9–76.1)	90.7 (87.6–93.8)	76.49 (0.011)	0.0 (0.6545)
Hcp1	4	66.3 (61.8–70.7)	94.2 (92.9–95.6)	87.08 (<0.0001)	91.07 (<0.0001)
OPS	3	59.8 (54.9–64.7)	93.4 (91.9–94.9)	87.61 (<0.0001)	95.67 (<0.0001)
EPS	4	75.1 (70.3–79.8)	79.2 (76.3–82.0)	74.34 (0.0038)	84.96 (0.0002)

Notes: IgG: immunoglobulin G; IgM: immunoglobulin M; ELISA: enzyme-linked immunosorbent assay; OmpA: outer membrane protein A; Hcp1: haemolysin co-regulated protein 1; OPS: O-polysaccharide; EPS: exopolysaccharide; CI: confidence interval
